# Major challenges and barriers in clinical decision-making as perceived by emergency medical services personnel: a qualitative content analysis

**DOI:** 10.1186/s12873-021-00408-4

**Published:** 2021-01-19

**Authors:** Mostafa Bijani, Saeed Abedi, Shahnaz Karimi, Banafsheh Tehranineshat

**Affiliations:** 1 Department of Medical Surgical Nursing, Fasa University of Medical Sciences, Fasa, 81936-13119 Iran; 2 Community-based Psychiatric Care Research Center, Department of Nursing, School of Nursing and Midwifery, Shiraz University of Medical Sciences, Shiraz, Iran

**Keywords:** Clinical decision-making, Emergency medical services, Qualitative research

## Abstract

**Background:**

Having to work in unpredictable and critical conditions, emergency care services (EMS) personnel experience complicated situations at the scene of accidents which, inevitably, influence their clinical decisions. There is a lack of research into the challenges which these professionals encounter. Accordingly, the present study aims to explore the major challenges and barriers which affect clinical decision-making from the perspective of EMS personnel.

**Methods:**

The present study is a qualitative work with a content analysis approach. Selected via purposeful sampling, the subjects were 25 members of the EMS personnel in Iran who met the inclusion criteria. The study lasted from December 2019 to July 2020. Sampling was maintained to the point of data saturation. Data were collected using semi-structured, in-depth, individual interviews. The collected data were analyzed via qualitative content analysis.

**Results:**

The results of data analysis were categorized into four themes and eight categories. The main themes were professional capabilities, occupational and environmental factors, inefficient organizational management, and ethical issues.

**Conclusion:**

The results of the present study show that clinical knowledge, experience, and skills contribute to emergency care personnel’s professional capabilities in making clinical decisions. Good teamwork skills and time management can prevent feelings of confusion when the number of the injured to be attended to is large. Effective clinical decision-making skills can not only help the personnel make the right decision, but enhances their resilience and enables them to adapt to hard and unpredictable conditions. Professional factors, organizational management, and ethical matters constitute the other major factors which influence the clinical decision-making of emergency care personnel at the scene of accidents and determine the quality of their clinical performance. Thus, it is essential that pre-hospital emergency care managers improve the quality of EMS personnel’s clinical decision-making skill.

**Supplementary Information:**

The online version contains supplementary material available at 10.1186/s12873-021-00408-4.

## Background

In most societies today, quality pre-hospital emergency care is an essential component of caring for patients in need of emergency care [1]. Pre-hospital emergency care consists of all the emergency medical services which are provided to patients outside the hospital before they are transferred to the nearest medical center [2]. In pre-hospital emergency care, seconds and minutes can mean the difference between life and death, between suffering a serious disability and living a normal life. In order for EMS personnel to provide timely care and evaluate patients’ status in the shortest possible time, it is necessary that they be equipped with effective clinical decision-making skills and be able to make the right decision for a patient in unpredictable conditions [3, 4]. Clinical decision-making, an important process in which the best action to achieve the desired goals is chosen, largely determines the quality of care, patient safety, and the possibility of future complications [5]. As an essential part of the professional duties of the medical personnel, clinical decision-making consists of analysis of information, making decisions, and taking action based on those decisions to accomplish the desired objective [6]. According to Emergency Nurses Association, clinical decision-making is one of the most important professional capabilities in emergency care personnel which can significantly improve the efficacy and quality of care provided [7].

The work environment in pre-hospital emergency care is complicated and unpredictable. At times, the EMS personnel should provide care to patients whose conditions are critical and unstable and who are in a life-threatening situation. Obviously, in order to make an appropriate response to quick changes in a patient’s conditions, EMS personnel should be able to decide quickly: they should know how to perform a quick evaluation of a patient’s status, determine the clinical priorities, and implement the correct intervention accordingly [8, 9]. If EMS personnel do not have good decision-making skills, they cannot make good clinical decisions and make the right intervention, which can make a patient’s condition more critical or, in some cases, even cause permanent injuries or death [10].

### Background in Iran

Pre-hospital emergency care in Iran dates back to 1975 when the first emergency care department was founded in the capital city of Tehran. After that, pre-hospital emergency care departments were gradually formed in the other provinces of Iran. These departments consist of urban centers and road centers. Pre-hospital emergency care personnel have an associate or bachelor’s degree in emergency care. Also, nurses who have a bachelor’s or master’s degree may be employed in pre-hospital emergency care. Two staff members must be present in every shift. Due to lack of staff, the shifts are on a 24-h basis and each staff member should work three 24-h shifts a week. Moreover, currently, only males are employed in pre-hospital emergency care and there is not any female staff. In pre-hospital emergency care departments, there is not a single set of clinical guidelines for the personnel and, in certain cases, the personnel act according to the internal regulations laid down by their mangers, which limits their freedom and independence.

Not many qualitative studies have addressed the factors which affect the clinical decision-making of emergency care personnel. However, a qualitative approach can result in an in-depth understanding of the concept in question and help determine its various aspects. Qualitative studies allow for determining the dimensions of a concept in a cultural context from the viewpoint of individuals who have been involved with the concept in question for a long time [11]. In addition, a qualitative approach can prove useful in identifying the barriers to clinical decision-making by emergency care personnel and achieving a deeper understanding of their experiences and challenges. In order for the quality of pre-hospital care to be improved, the rich experiences of the personnel with regard to the challenges of clinical decision-making need to be addressed. Without an understanding of the feelings and challenges which emergency care personnel experience during clinical decision-making at the scene of accidents, it is not possible to develop their professional capabilities and improve the quality of pre-hospital emergency care. Accordingly, the present study is a qualitative attempt at investigating the leading challenges and barriers in EMS personnel’s clinical decision-making.

## Methods

The present study is a qualitative work of research with a content analysis approach. Conventional content analysis in qualitative research is applied when there is a lack of knowledge about the concept under study. Since the body of literature on the leading challenges and barriers in EMS personnel’s clinical decision-making is quite small, the researchers used the above-mentioned approach for their study [12]. Twenty-five members of pre-hospital emergency care personnel were selected via purposeful sampling and invited to participate in the study. Purposeful sampling is one of the frequently-used methods in qualitative studies. In purposeful sampling, the subjects are selected by the researcher. The subjects who are selected have rich and in-depth knowledge and experience of the concept under study and can provide a detailed insight into it [13]. Thus, the researchers selected individuals with extensive experience or knowledge of the subject of the study. After consulting the emergency care mangers, the researchers selected one of the personnel who could interact better and provide rich information for the initial interview. Then the first participant was asked to name a colleague who had rich knowledge and experience of the subject under study. In this manner, based on the information provided by the mangers and the participants chosen earlier, more participants who had a good understanding of the subject of the study were selected. Sampling was maintained to the point of data saturation. Data saturation was achieved when no new data could be collected and no new categories or sub-categories could be extracted [14]. In this study, data saturation was reached after 23 interviews, and two further interviews were done to ensure no new data was available.

Yet, to ensure that the data was saturated, the researchers conducted two additional interviews. The inclusion criteria were having at least 1 year’s experience of work in pre-hospital emergency care, being willing to participate in the study, and being able to provide extensive and rich information about the challenges and barriers in clinical decision-making.

Data were collected through semi-structured, individual interviews, in the interval between December 2019 and July 2020. All the interviews were conducted by the first author (MB) in the conference room of the emergency department after prior arrangements with the head of the emergency department and the participants. Each interview lasted from 45 to 60 min.

The interview guide was developed according to the views of the research team and the available literature. The face and content validity of the questionnaire was examined and verified by 10 members of the emergency care personnel and 4 pre-hospital emergency care experts and professors. Accordingly, the face validity of the interview guide was determined based on the results of three initial interviews with the participants and some minor changes were made to make the questions more comprehensible. The content of the three interviews was not included in the final analysis. The focuses of interview questions were based on your experiences; what factors can influence clinical decision-making?” What skills do you need to make the right clinical decision? (Additional file 1: Interview Guide and Question).

At the end of each interview, the participants were thanked for their cooperation and were asked for their phone numbers in case the interviewer had any other questions, needed the participants to confirm their statements, or had to interview a participant again. Immediately after completion, each interview was transcribed. To maintain confidentiality with regard to the information acquired from the participants by phone or in interviews, the researchers assured the participants that their information would be used for research purposes only and would remain confidential throughout the study and after in the process of collecting and analyzing the data.

The collected data were analyzed using Graneheim and Lundman’s approach to content analysis (2004): initially, to immerse in the data and to achieve a general idea of it, the researchers read each transcript several times. Next, the words, sentences, or paragraphs which carried significance were selected as meaning units. The meaning units were assigned codes which showed a summary of the meaning of the unit and then the texts were codified. Afterwards, the codes were compared in terms of their similarities and differences, the similar codes were merged, and the codes and texts were reviewed again. Based on their similarities, the meaning units were categorized. To ensure the reliability of the codes, the researchers reviewed the categories and compared them against the data again. The themes were identified after deep reflection and comparison of the categories with each other. In this regard we try to use both manifest and latent analysis [15]. Accordingly, after completion, each interview was immediately transcribed by the first author (MB). Subsequently, the first author read and re-read the transcripts and, after obtaining a general understanding of the content, analyzed the data inductively. When the transcripts were being read, significant paragraphs and sentences were marked. The words, sentences, and paragraphs which contained a significant point about the challenges of clinical decision-making were selected as semantic units. Each significant paragraph or phrase was assigned a code. In the next stage, the third author (SK) reviewed the transcripts and verified the semantic units and open codes. Possible disagreements over the semantic units and codes were resolved in meetings attended by all the research team members (MB, SK, SA, and BT). The research team also verified the quality of data analysis. Based on the similarities and compatibilities between them, the codes were classified and categories were developed. To ensure the reliability of the codes, the researchers reviewed the categories and compared them with the data again. Eventually, in consecutive, joint meetings, the research team extracted the main themes after careful consideration and comparison of the categories. The collected data were analyzed in MAXQDA v. 2007 (Table 1, Example of data analysis process).

**Table 1:** An example of coding and development of categories and themes

Meaning units	Coding	Category	Theme
On some missions when we have to deal with a large number of casualties, my colleagues and I get confused and find it hard to make a good decision. In my experience, this is because we are not skilled enough in teamwork and time management (P,19)	Teamwork skill Time management	Clinical competence	Professional capabilities
Some of the ambulances in inter-city and rural emergency stations lack such medical devices as suction machines, cardiac monitors, pulse oximeter, and ventilators. Even now that we have to deal with the COVID-19 epidemic, we don’t have access to personal protective equipment, like masks, gloves, and special gowns (P,24)	Lack of medical devices Lack of personal protective equipment	Challenges of Human resources and medical equipment	Inefficient organizational management

To ensure the trustworthiness of the results, the researchers applied Lincoln and Guba’s criteria [16]. Accordingly, to ensure credibility, the researchers applied prolonged engagement with data, member checking, and peer debriefing. For member-checking, 2 of the pre-hospital emergency care personnel were presented with a copy of the encoded interviews which was confirmed. To perform peer-checking, the researchers had four experts analyze and observe the process of data analysis and validate the codes and categories. The dependability of the study results was enhanced through describing the methods used to code the concepts and themes, as well as presenting textual and audio data. Also, two members of the research team examined the findings individually and then discussed them to resolve any possible disagreements to ensure dependability. To ensure that the results were confirmable, the researchers showed the encoded data to the participants to verify the accuracy of the extracted categories and subcategories. The conditions of the respondents and how they were interviewed were clearly described to add to the transferability of the study results. The researchers also tried to select the study subjects solely based on the objectives of the study and free of bias. Data were analyzed as soon as they were collected to help the researcher be aware of the principles of the research. Transferability was ensured by providing a comprehensive description of the subject of the study, the participants’ characteristics, and methods of data collection and data analysis, along with documented examples of the participants’ quotes.

In the process of collecting and analyzing the data, the researchers applied bracketing. Bracketing is a method used in qualitative research to minimize the potential threats of prejudgment which can adversely affect the research process [17]. Thus, the researchers tried to ignore their own personal knowledge, beliefs, values, and experiences in order to accurately describe the emergency care personnel’s views on the challenges of clinical decision-making. The researchers did not judge the data and accepted it as it was.

### Ethical considerations

All participants gave written informed consent to participate in the study. The present study was conducted in accordance with the principles of the revised Declaration of Helsinki, a statement of ethical principles which directs physicians and other participants in medical research involving human subjects. Moreover, the study was approved by the local Ethics Committee of Fasa University of Medical Sciences, Fasa, Iran (IR.FUMS.REC.1398.168).

## Results

This study recruited a total of 25 subjects. The mean and standard deviation of the participants’ ages was 33.52 ± 5.89 years. The personal characteristics of the participants are shown in Table 2. Based on the participants’ experiences, as a major professional capability which emergency care personnel should be skilled at, clinical decision-making is comprised of clinical knowledge, skills, experience, and judgment for identifying patients’ problems, analyzing their conditions, making the right decision, and taking effective measures based on that decision. From the participants’ point of view, clinical decision-making is influenced by a wide variety of factors. Analyses of the qualitative data yielded four themes and eight categories (Table 3).

**Table 2:** Individual characteristics of the participants

Variable	Mean ± SD / frequency (number)
Work experience	8.40 ± 4.83 years
Education level	
Associate’s degree in EMS	24 (6)
Bachelor’s degree in EMS	56 (14)
Bachelor’s degree in nursing	16 (4)
Master’s degree in nursing	4 (1)

**Table 3:** Themes and categories extracted from content analysis

Theme	Categories	Selective Coding
Professional capabilities	Clinical Competence	• Clinical knowledge • Clinical experience • Clinical skills • Teamwork skills • Time management skills • Clinical judgment skills
	Psychological empowerment	• Emotional stability • Resilience • Anger management
Occupational and environmental factors	Occupational factors	• Time of missions • Location of missions • Patients’ status • Fatigue and occupational burnout
	Environmental factors	• Conditions at the scene of accidents • Spread of infectious diseases
Inefficient organizational management	Structural Challenges	• Lack of a counseling doctor at the emergency dispatch centers • Conflicts in the regulations • lack of a clear operations manual
	Challenges of Human resources and medical equipment	• Shortage of medical equipment • Lack of personal protective equipment • Staff shortages
Ethical issues	physical privacy	• Respect for patients’ physical privacy • Respect for patients’ sexual privacy • Respect for patients’ religious beliefs
	sexual privacy	

### Professional capabilities

“Professional capabilities” was the most noticeable theme extracted from the participants’ experiences. This theme consists of the following categories: Clinical Competence and Psychological empowerment consists of emotional stability and resilience.

#### Clinical competence

Clinical Competence consists of clinical knowledge, clinical experience, clinical skills, teamwork skills, time management skills and clinical judgment skills.

According to one of the participants:

*One of the most important professional capabilities that emergency care personnel need to make quick and correct clinical decisions is clinical knowledge. Unfortunately, some of our colleagues here don’t have enough clinical knowledge. I mean, for example, they don’t know much about the physiopathology of diseases. How can you possibly make a good clinical decision and take effective clinical measures if you are ignorant about the causes and treatments of diseases? (P10, 18 years of experience)*

Another participant stated that:

*Some of our colleagues have a lot of clinical experience and can make a quick diagnosis at first glance at a patient and then make the right clinical decision accordingly. (P3, 16 years of experience)*

Another subcategory of professional capabilities which was found to have an impact on clinical decision-making was clinical skills. One of the participants remarked:

*There are people here who don’t have the required clinical skills. For instance, they can’t do a proper assessment of a patient’s status, or they don’t know how to use the equipment. On many occasions, when we were on a mission, one of my co-workers couldn’t use the cardiac monitoring device or the defibrillator the right way. If you can’t do a proper monitoring or assess your patients, how can you make the right clinical decision and do the right thing for them? (P7, 9 years of experience)*

“Teamwork skills” was another component of professional capabilities referred to by many of the participants. Based on the participants’ experiences, having good teamwork skills can contribute to good decision-making and, as a result, providing quality care. Sometimes, the emergency care personnel encounter a large number of patients or casualties and need to possess satisfactory teamwork skills and time management skills to make good clinical decisions. According to one of the participants:

*On some missions when we have to deal with a large number of casualties, my colleagues and I get confused and find it hard to make a good decision. In my experience, this is because we are not skilled enough in teamwork and time management. At the scene of accidents, everyone wants to be boss and tell the others what to do and that causes chaos which wastes our time and puts a patient’s life at risk. (P19, 12 years of experience)*

Another aspect of professional capabilities with an impact on clinical decision-making is clinical judgment. From the participants’ experiences, good clinical judgment plays an important part in making correct and reasonable clinical decisions in all situations, especially in emergency situations where a quick diagnosis is necessary. As one of the participants pointed out:

*A skillful and capable member of the emergency care personnel should be able to analyze and interpret the implicit and explicit symptoms of a patient and use that information to make a proper clinical judgment and clinical decision. (P12, 15 years of experience)*

Another participant stated that:

*One of the major challenges and problems which I have repeatedly seen in the clinical decision-making of the emergency care personnel is poor clinical judgment. Some of my co-workers don’t have the necessary skills in this area and can’t come to a good conclusion and decision based on the status of a patient and analysis of their symptoms. And so they make mistakes in their decision-making and I’ve sometimes seen them put a patient’s life in danger. (P8, 10 years of experience)*

#### Psychological empowerment

Resilience is another component part of Psychological empowerment which the participants’ experiences showed to affect clinical decision-making. From the participants’ point of view, resilience means that the emergency care personnel should be flexible, should be able to adapt to the hard and unpredictable conditions of work in pre-hospital emergency care, and should possess great tolerance. One of the participants stated that:

*Having a high tolerance threshold is an essential quality in pre-hospital emergency care personnel. I’ve witnessed cases where some personnel with good professional knowledge and skills lost their patience in critical conditions or when the number of the injured was high. They couldn’t manage the scene of the accident and make a logical clinical decision. (P16,14 years of experience)*

Another component of Psychological empowerment which has an impact on clinical decision-making is emotional stability. According to one of the participants:

*Work conditions in pre-hospital emergency care are very complicated and unpredictable. So it is necessary for emergency care personnel to be emotionally stable so they can manage the scenes of accidents well, keep calm, make the right decision, and take effective clinical measures. (P25, 2 years of experience)*

Another participant remarked that:

*Unfortunately, some of my colleagues do not have emotional stability: they lose their temper easily and can’t manage their anger. Sometimes we come across patients who are very aggressive and irritable. If we don’t manage our feelings and emotions, we can’t make the right decision for them and may even put their lives in danger. (P2, 10 years of experience)*

### Occupational and environmental factors

Another theme of the major challenges and barriers which affect clinical decision-making is occupational and environmental factors. This theme consists of the following categories: Occupational factors and Environmental factors.

#### Occupational factors

Occupational factors consist of time of missions, location of missions, Fatigue and occupational burnout and patients’ status.

One of the occupational factors referred to by the participants is the time of missions. At times, pre-hospital emergency care personnel have to go on a mission at night and in complete darkness or in bad weather conditions, which circumstances can adversely affect the speed and accuracy of the personnel’s decision-making. According to one of the participants:

*Many times, I’ve had to be present at the side of a patient in unstable weather conditions, in foggy weather, or at night. It is really hard to work in such circumstances and to judge the situation and make the right clinical decision quickly. I would rather go on a hundred missions in one shift at day time than get dispatched at night or in bad weather. (P5, 8 years of experience)*

Another occupational factor from the participants’ perspective is the location of missions in pre-hospital emergency care. One of the participants mentioned that:

*Serving in inter-city stations and rural areas is much harder and more stressful than working in stations located in the city. Since we have to drive long distances and it takes much time to get to urban medical centers, when patients’ conditions are critical, we really get confused and can’t make the right decision immediately and provide the necessary care. (P9, 8 years of experience)*

Patients’ status and the conditions at the scene of accidents are other factors which affect clinical decision-making. According to one of the participants:

*Sometimes, the scene of an accident is very unsafe and full of hazards and these conditions have a negative impact on our decision-making. For instance, I’ve occasionally had to appear at the scene of a murder or shooting incident, or where there was an ongoing fight, or where a house or a vehicle was on fire. Well, under such unsafe circumstances, how am I supposed to care for a patient when my own life is in danger? (P11, 5years of experience)*

Patients’ status one another of the occupational factors which affect clinical decision-making is emergency care.

Another participant stated that:

*Our decisions are also affected by the clinical conditions of a patient. For example, sometimes, the patient is unconscious or the patient is deaf or speech-impaired and can’t communicate with us. At such times, it is not possible to acquire reliable data about a patient’s status and make the right decision. (P6, 7 years of experience)*

Another of the occupational factors which affect clinical decision-making is emergency care personnel’s fatigue and occupational burnout. As one of the participants remarked:

*Working in pre-hospital emergency care is really demanding and there are not many who can bear the pressure. Sometimes, I’ve had to go on about 30 missions in a 24-hour shift. Work overload and shortage of experienced staff have caused fatigue and burnout in the personnel and these have negative effects on our concentration, mental acuity, and decision-making. (P13, 4 years of experience)*

#### Environmental factors

Environmental factors consist of conditions at the scene of accidents and spread of infectious diseases. According to one of the participants:

“Sometimes, the scene of an accident is very unsafe and full of hazards and these conditions have a negative impact on our decision-making. For instance, I’ve occasionally had to appear at the scene of a murder or shooting incident, or where there was an ongoing fight, or where a house or a vehicle was on fire. Well, under such unsafe circumstances, how am I supposed to care for a patient when my own life is in danger?” (P11, 5 years of experience)

Spread of infectious diseases is another dimension of the environmental factors. According to one of the participants:

*When there is an outbreak of a dangerous infectious disease, like Ebola, H1N1, or COVID-19, sometimes there is a conflict between the emergency care personnel’s decisions and what the patients want. Even if we judge a patient’s condition to be critical and decide that he or she should be transferred to the hospital, the patient or the patient’s family don’t care about our decision and refuse to have the patient transferred because of their fear of the epidemic. (P15, 3 years of experience)*

On a similar note, another participant stated that:

*On a mission, I had to give care to a patient who had symptoms of sudden cardiac arrest. Even though I told the patient and his family that the patient’s condition was serious, they ignored my decision and said because of the spread of the coronavirus, they wouldn’t have their patient taken to the hospital. (P17, 5 years of experience)*

### Inefficient organizational management

Another theme of the major challenges and barriers in clinical decision-making is inefficient organizational management which consists of the following categories: Structural challenges, and challenges of human resources and medical equipment.

#### Structural challenges

Structural challenges consist of management’s failure to support the personnel in the case of errors in decision-making and legal troubles, inefficiency of the dispatch center, management’s failure to conduct root cause analysis and take corrective action in the case of errors in decision-making, conflicts in the regulations and lack of a clear operation manual.

Another component part of structural challenges from the participants’ perspective is the managers’ failure to support the personnel in the case of errors in decision-making and legal troubles. Many of the participants stated that they are not adequately supported by their mangers if legal issues arise and the patients press charges against the personnel. They also mentioned that the mangers do not take any effective measures toward analyzing the root causes of clinical errors and or take corrective action.

According to one of the participants:

*Unfortunately, in matters of legal issues and charges, the managers don’t support the personnel as much as they’re expected to. There are many cases in which, because of a clinical error, patients’ families have sued the personnel, but the management failed to give enough support. If you do a good job on a hundred tasks, they will tell you that you are just doing your duties. But if you make a single mistake, you’ll immediately get reprimanded. Out of this fear of being reprimanded or sued by patients, some of the personnel do not report the errors in their clinical decision-making. (P24, 11 years of experience)*

Conflicts in the regulations and lack of a clear operations manual” was another issue referred by the participants. In this relation, one of the participants stated that:

*There is not a clear manual which describes the responsibilities of the emergency care personnel. For instance, according to the internal codes, the pre-hospital emergency care personnel should not administer intravenous medications and if they do and their action puts a patient’s life in danger, they will be held liable. Sometimes, we really don’t know what the right decision is and what kind of action we should take. When a patient is having a seizure, should I administer diazepam to them or not? (P20, 15 years of experience)*

Another participant remarked that:

*Once, I had a 45-year old patient with severe chest pains. Based on his symptoms, my diagnosis was cardiac arrest and we transferred him to the hospital. In the triage unit, the emergency medicine doctor scolded me for not having given an intravenous nitroglycerin injection to the patient. He said all we know is how to transfer patients! I explained to him that we’re not allowed to administer medicine intravenously. (P1, 11 years of experience)*

Inefficiency of the dispatch center is another aspect of the structural challenges referred by the participants. One of the participants’ comments in this relation is as follows*:*

*The dispatch center is the brains and director of the operations in pre-hospital emergency care. Unfortunately, employment of individuals who lack the required knowledge and skills in this field disrupts the operations. For instance, one time, the personnel at the dispatch unit announced that a patient had signs of weakness and dizziness and could have hypoglycemia. When we got to the patient’s side, we realized he’d had cardiac arrest and that the dispatch center had suggested hypoglycemia by mistake. Misleading information from the dispatch unit can prevent the personnel from making the right diagnosis and, consequently, the right decision. (P4, 2 years of experience)*

#### Challenges of human resources and medical equipment

Challenges of human resources and medical equipment consist of employment of inexperienced and inefficient personnel, shortage of equipment and not organizing workshops for the professional empowerment.

Employing personnel who don’t have the necessary expertise and skills in pre-hospital emergency care is another component part of challenges of human resources and medical equipment which the participants’ experiences showed to affect clinical decision-making.

One of the participants’ comments in this relation is as follows:

Employing personnel who don’t have the necessary expertise and skills in pre-hospital emergency care is actually gambling with people’s lives. Unfortunately, because of organizational benefits and lack of budget, the senior managers hire inexperienced individuals who don’t have the necessary knowledge and skills in pre-hospital emergency care. On many occasions, I’ve seen some of my colleagues who lack expertise in making a diagnosis make wrong decisions and put a patient’s life in danger. When we object to the mangers, they say the budget is limited and they can’t afford to hire skilled workforce. (P23, 17 years of experience).

Not organizing workshops for the professional empowerment is another component part of challenges of human resources and medical equipment which the participants’ experiences showed to affect clinical decision-making.

According to one of the participants:

*Unfortunately, the administration does not take any special measures to update the knowledge and skills of the personnel. Over the past there, not a single workshop has been held and the knowledge of most of the personnel is not up-to-date. We have repeatedly asked for training courses in basic and advanced CPR and trauma emergency care, but the administration says it doesn’t have any budget for workshops. How can the personnel make good clinical decisions and do the right thing for their patients when their knowledge and skills are not up-to-date? (P10, 18 years of experience)*

Another issue under the category of challenges of human resources and medical equipment is shortage of equipment. One of the participants stated that:

*Some of the ambulances in inter-city and rural emergency stations lack such medical devices as suction machines, cardiac monitors, pulse oximeter, and ventilators. Even now that we have to deal with the COVID-19 epidemic, we don’t have access to personal protective equipment, like masks, gloves, and special gowns, and our fear of getting the infection has a negative effect on our diagnoses and, in turn, our decisions and actions. (P24, 13 years of experience)*

According to another participant:

*When there is no heart monitor, pulse oximeter, or ventilator inside the ambulance, how am I supposed to diagnose a patient’s cardiac or respiratory problem and make a proper clinical decision that won’t be a threat to the patient’s life? (P18, 6 years of experience)*

### Ethical issues

The final theme of the major challenges and barriers in clinical decision-making is “ethical issues” which consists of categories of respect for patients’ physical privacy and respect for patients’ sexual privacy.

#### Respect for patients’ physical privacy

Regarding respect for patients’ physical privacy, in the Iranian culture, it is important that patients’ physical privacy be maintained and that their private parts not seen by caregivers, especially opposite-sex caregivers. According to a participant:

*Sometimes, we must provide care to female patients, especially young females, who have palpitations or chest pains and are in need of immediate cardiac monitoring. It is not ethical for the patients or even for us who are in the Iranian culture to see the female patient’s body. It is really a tough job to make a decision at such times and the patients may get very uncomfortable. (P13, 4 years of experience)*

#### Respect for patients’ sexual privacy

The participants also mentioned that, considering the dominant Islamic culture in Iran, showing respect for the sexual privacy of patients is very important to patients and their families. One of the participants stated that:

*While we were transferring a pregnant woman, we realized she had broken her water and was in labor. The baby was coming and we had to help her deliver it. The poor woman seemed very embarrassed and uncomfortable. It is so hard to make a decision in such conditions. After all, we are Muslim and our ethical principles dictate that at such times, care should be given by a person of the same gender as the patient. But in Iran, there are no female staff members in the pre-hospital emergency care*. *(P21, 3 years of experience)*

## Discussion

This study explored the major challenges and barriers which affect clinical decision-making from the perspective of EMS personnel. The analysis of the participants’ experiences showed that challenges and barriers of the participants’ clinical decision-making consisted of four areas: professional capabilities, occupational and environmental factors, inefficient organizational management, and ethical issues.

At the scene of accidents, the emergency care personnel encounter various clinical challenges which affect their clinical decision-making [18]. At the same time, it is the ethical and professional responsibility of emergency care personnel to provide quality care based on code of ethics to victims at the scene of accidents [19]. The findings of the present study concern pre-hospital emergency care personnel’s perception of the major challenges which affect clinical decision-making at the scene of accidents.

Professional capabilities, including clinical knowledge and skills, were found to play an important part in pre-hospital emergency care personnel decision-making. Similarly, in their study of the barriers to nurses’ clinical decision-making in the university hospitals of the south of Iran, Mousavi et al., report that having knowledge, clinical experience, and clinical skills are essential to making good clinical decisions—the clinical decision-making mean score of the nurses who had poor clinical knowledge, experience, and skills was unsatisfactory [20]. Another important component of professional capabilities which has a significant impact on clinical decision-making is clinical judgment. According to the study of Engebretsen et al., clinical judgment is a necessary skill for all nurses, especially pre-hospital emergency care nurses, as it enables them to make accurate diagnoses and make proper clinical decisions [21]. Likewise, the results of the study of Anderson et al., show that clinical judgment and critical thinking play a key role in making clinical decisions in all situations, in particular the critical and complicated conditions which prevail in pre-hospital emergency care [22]. In the present study, the participants stated that some of their colleagues do not have adequate clinical knowledge, skills, and experience and cannot, therefore, make the right decision for their patients. Similarly, the results of the study of Perona et al., show that clinical knowledge and skills and clinical experience are key factors in emergency care personnel’s clinical decision-making. Lack of those capabilities poses a challenge when the personnel should make clinical decisions and adversely affects the quality and safety of the care. Accordingly, it is necessary for pre-hospital emergency care administrators to take measures to improve the professional capabilities of their personnel [23]. Emergency Nurses Association (ENA) has always stressed that advances in technology, increase in professional knowledge, and work specialization mean that emergency care nurses’ clinical knowledge and performance need to be improved regularly. Since emergency care is an unpredictable area and, occasionally, the personnel have to deal with a large number of patients with various issues, it is necessary for the emergency care personnel to possess such professional capabilities as clinical knowledge and skills to be able to make the right clinical decision and provide safe and quality care accordingly [24]. The results of the study of Aloyce, et al. show that the emergency nurses who lack the required clinical knowledge and skills make mistakes in their clinical decision-making and triage [25].

In the present study, “teamwork skills” was found to be another important aspect of professional capabilities which affects clinical decision-making. In Grover’s qualitative-exploratory study of emergency nurses’ experiences of teamwork in a hospital in Australia, the participants stated that teamwork skills are among the essential clinical skills which emergency nurses must possess. The participants’ experiences showed that when the emergency department is busy and a large number of patients must be attended to, teamwork skills can significantly facilitate clinical decision-making. The personnel with poor teamwork skills get nervous and confused at such times and cannot make logical decisions for their patients [26].

Based on the participants’ experiences, the emergency care personnel occasionally have to provide care to patients who are very irritable and aggressive. If the personnel fail to manage their own feelings and emotions in such circumstances, they cannot make a good clinical decision for the patients and may even take actions which put the patients’ lives at risk. The results of the study of Lin et al., show that emergency nurses must possess high levels of resilience and tolerance so that they can employ their capabilities in the critical conditions of emergency situations and, by making quick and correct clinical decisions, implement effective clinical interventions [27]. Halpern, states that pre-hospital emergency is a complicated, unpredictable, and stressful environment. At times, patients or their companions, who are under considerable stress, may behave in violent and insulting ways. Therefore, it is essential for the emergency care personnel to possess such psychological qualities as resilience and emotional intelligence to be able to control their emotions and make the right clinical decision and, consequently, take effective clinical measures. By holding workshops, pre-hospital emergency care administrators can enhance the personnel’s psychological capabilities [28].. Similarly, Gunnarsson et al., report that emergency nurses must possess resilience and emotional stability to be able to use their professional abilities effectively and take proper clinical measures at the time of an emergency. Although such factors as fatigue from work overload, shortage of personnel, and stressful work conditions in emergency departments have an adverse effect on the performance of emergency care personnel, nurses who are resilient and can manage their emotions continue to provide high-quality care [29].

Occupational and environmental factors constitute another category of the major challenges and barriers which affect emergency care personnel’ clinical decision-making. According to the study of Li, et al. (2018), shortage of experienced nurses in emergency departments, increase in workload, and fatigue and burnout are the most significant occupational factors which adversely affect the personnel’ diagnoses and decision-making and lead to patients’ dissatisfaction and poor-quality care [30].

Another category of the major challenges which affect emergency care personnel’ clinical decision-making is inefficient organizational management. According to the study of Bijani et al., employment of inexperienced personnel who lack the required expertise, lack of facilities and equipment, inadequate support for the personnel, absence of clear instructions, conflicts in the regulations, and failure to hold workshops for the professional empowerment of emergency nurses are the most significant challenges which affect the quality of triage in emergency departments [31]. These findings are consistent with the results of the present study.

From the participants’ perspective, lack of an emergency doctor to consult and lack of a set of standard clinical guidelines are among the major barriers to satisfactory clinical decision-making. Thus, it is recommended that the senior management at emergency care departments set standard evidence-based clinical guidelines for the personnel to follow. Also, pre-hospital emergency care personnel should have access to the clinical consultation of an emergency doctor stationed at the emergency care center. Likewise, Bashiri et al. report that evidence-based clinical guidelines and instructions and having access to advice from a doctor in emergency care departments play a key role in improving the process of clinical decision-making by emergency care personnel and will result in higher quality care [32].

Another important category which was found to affect emergency care personnel’ clinical decision-making is ethical issues. Showing respect for the privacy of patients and maintaining their human dignity are among the most important ethical responsibilities of caregivers [33]. Accordingly, all caregivers are expected to maintain their patients’ privacy and dignity [34].

In the present study, too, the views of the interviewed pre-hospital emergency care personnel showed that one of the key factors which influence clinical decision-making is showing respect for the physical and sexual privacy of patients. The dominant religious-cultural beliefs and values in Iran dictate that the bodies of all individuals, especially women, should be covered, in particular their genitalia and breast area, and that there should not be any physical contact between men and women who are not related to each other. Thus, to respect the privacy of patients and accident victims, even in pre-hospital emergency care, it is necessary that patients receive care from same-gender caregivers. But the national laws of Iran forbid women from working in pre-hospital emergency care. Therefore, there is need for changes which will allow the employment of female personnel in emergency care. On a similar note, the results of the study of Torabi et al., show that, from the viewpoint of pre-hospital emergency care personnel, maintaining the physical and sexual privacy of patients is among the key aspects of clinical and ethical decision-making [35].

In pre-hospital emergency care, maintaining patients’ privacy according to the patient’s physical conditions, personal life, and lifestyle is essential. These rights apply to deceased victims, too [36]. In the study of Abelsson & Lindwall, the participants state that covering the body of a victim, e.g. keeping a patient’s body covered when electrodes are being attached, is essential to maintaining his/her privacy. Keeping a victim’s body covered in line with the victim’s values and beliefs while emergency care is being given to him/her at the scene is an ethical obligation [37]. Maintaining patient privacy and patient dignity are closely connected. Dignity is a social and cultural concept and individuals’ perceptions of maintaining privacy and dignity are influenced by their beliefs and values [38]. As a result of the dominant religious and cultural beliefs in Iran, women feel embarrassed when they remove their clothing to be examined by a male nurse or doctor and consider that situation to be unethical. Therefore, EMS male personnel in Iran feel uncomfortable when they should provide services to female victims at the scene of accidents, but try to give care based on their cultural and religious values and commitment to professional ethics.

### Strengths of the study

The present study is the first qualitative attempt at exploring the challenges which affect clinical decision-making from Iranian pre-hospital emergency care personnel’s point of view and can be regarded as a significant accomplishment. In addition, in the present study, a wide range of the factors which have an impact on pre-hospital emergency care personnel’s clinical decision-making were identified. These findings can be employed to improve the quality of pre-hospital emergency care services and the personnel’s clinical decision-making skills.

### Limitations of the study

One of the limitations of the study is that it only addressed the views of pre-hospital emergency care personnel and the ideas of patients, as the beneficiaries of emergency care services, were not taken into account. Also, due to differences between the cultural, social, and economic conditions of Iran and those of other countries, the results may not be completely transferable and similar studies should be carried out in other countries.

## Conclusion

In today’s healthcare systems, patients in critical conditions usually receive their first care from pre-hospital emergency care personnel. The more correctly, accurately, and quickly these professionals act, the lower will be the rate of fatalities and possible disabilities and the more trust people can place in the services provided by pre-hospital emergency care departments. The success of these departments depends on a variety of factors, one of the most important of them being the ability to make good clinical decision-making. Therefore, it is necessary that those challenges should be identified and addressed in order for the quality of emergency services to improve. The results of the present study show that clinical knowledge, experience, and skills contribute to emergency care personnel’s professional capabilities in making clinical decisions. Good teamwork skills and time management can prevent feelings of confusion when the number of the injured to be attended to is large. Effective clinical decision-making skills can not only help the personnel make the right decision, but enhances their resilience and enables them to adapt to hard and unpredictable conditions. Professional factors, organizational management, and ethical matters constitute the other major factors which influence the clinical decision-making of emergency care personnel at the scene of accidents and determine the quality of their clinical performance. Thus, it is essential that pre-hospital emergency care managers improve the quality of EMS personnel’s clinical decision-making skill.

## Supplementary Information

**Additional file 1.** Interview Guide and Question.

## Data Availability

The datasets used and/or analysed during the current study are available from the corresponding author on reasonable request.

## Abbreviation

EMS: Emergency medical services
