# Supplementary material for: Major challenges and barriers in clinical decision-making as perceived by emergency medical services personnel: a qualitative content analysis
Source: BMC Emerg Med. 2021 Jan 19;21:11. doi: 10.1186/s12873-021-00408-4 (PMC7815282; doi:10.1186/s12873-021-00408-4)
Supplement: Supplementary file 1 — Additional file 1. Interview Guide and Question. [file 12873_2021_408_MOESM1_ESM.docx]

Additional file1: Interview Guide and Question

| Interview Guide  Thank you for accepting to be interviewed by us. The study we are undertaking is to understand more about the Major Challenges and Barriers in Clinical Decision-making as Perceived by Emergency Medical Services Personnel. I will be asking you several questions which are relevant to the study. You may respond to these queries in any way you feel comfortable. It is perfectly fine if you do not want to respond. At any point during the interview, if you are not clear about any questions, you are free to clarify the same with us and ask us to explain further. The information obtained during the interview will be kept confidential and will be shared only with the research team. We would like to audio record the interview in order to ensure that we do not miss out any salient issues. The recordings will be kept confidential. Your identity will be protected and your interview will also be labeled in codes. Is it OK with you that we audio record the interview?  Personal Information: Age:  Qualification: Associate's degree in EMS / Bachelor's degree in EMS/ Bachelor's degree in nursing/ Master's degree in nursing Months / Years of experience in EMS _________  **Interview questions**:  1. Based on your experience, what is your definition of clinical decision-making?”  2. “Based on your experience, what is the significance of clinical decision-making in pre-hospital emergency care services?”  3. “Based on your experience, can you suggest strategies to eliminate the challenges which affect clinical decision-making?”  4. What factors can influence clinical decision-making?  5. What skills do you need to make the right clinical decision?  6. Do your co-workers have the necessary skills for good clinical decision-making? |
| --- |
